# Ceramic Laser Materials

**DOI:** 10.3390/ma5020258

**Published:** 2012-02-09

**Authors:** Jasbinder Sanghera, Woohong Kim, Guillermo Villalobos, Brandon Shaw, Colin Baker, Jesse Frantz, Bryan Sadowski, Ishwar Aggarwal

**Affiliations:** 1 US Naval Research Lab, Washington, DC 20375, USA; E-Mails: guillermo.villalobos@nrl.navy.mil (G.V.); brandon.shaw@nrl.navy.mil (B.S.); colin.baker@nrl.navy.mil (C.B.); jesse.frantz@nrl.navy.mil (J.F.); 2 Sotera Defense Solutions, Crofton, MD 21114, USA; E-Mails: bryan.sadowski.ctr@nrl.navy.mil (B.S.); ishaggar@nrl.navy.mil (I.A.)

**Keywords:** ceramics, laser materials, 100 KW, microchip lasers, ultrashort pulse, ceramic composites, non-oxide ceramics

## Abstract

Ceramic laser materials have come a long way since the first demonstration of lasing in 1964. Improvements in powder synthesis and ceramic sintering as well as novel ideas have led to notable achievements. These include the first Nd:yttrium aluminum garnet (YAG) ceramic laser in 1995, breaking the 1 KW mark in 2002 and then the remarkable demonstration of more than 100 KW output power from a YAG ceramic laser system in 2009. Additional developments have included highly doped microchip lasers, ultrashort pulse lasers, novel materials such as sesquioxides, fluoride ceramic lasers, selenide ceramic lasers in the 2 to 3 μm region, composite ceramic lasers for better thermal management, and single crystal lasers derived from polycrystalline ceramics. This paper highlights some of these notable achievements.

## 1. Introduction 

**Figure 1:**
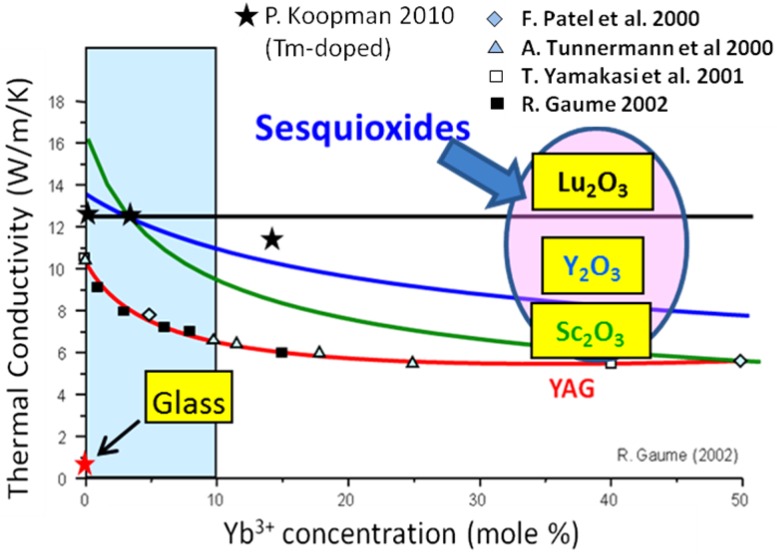
Thermal conductivity *versus* dopant concentration for crystals (Adapted from R. Gaume 2002 [4], P. Koopmann [6], F. Patel [7], A. Tunnermann [8] and T. Yamakasi [9]).

Solid state lasers are advantageous over gas lasers and free-electron lasers due to significantly smaller footprints, potential for enhanced mobility and excellent performance. Examples of these include slab, rod and disk type of lasers based on rare earth doped crystals. Currently, rare earth doped yttrium aluminum garnet (YAG), is the most extensively studied and widely used for high power lasers. An example is the Nd^3+^:YAG (yttrium aluminum garnet) laser which is based on a single crystal of YAG doped with Nd^3+ ^ions. More recently, ceramic Nd^3+^:YAG has been fabricated and used to demonstrate 67 kW [1] and >100 kW [2] of output power at 1.06 μm, respectively. However, YAG is not the best host material for high-power laser operation systems due to its relatively low thermal conductivity and high thermal expansion. The sesquioxides such as Sc_2_O_3_, Y_2_O_3_, and Lu_2_O_3_ are very promising host materials for high-power laser applications, mainly due to their high thermal conductivity and high absorption and emission cross-sections of trivalent rare-earth ions in these materials [3,4] (Figure 1). Among them, Lu_2_O_3_ stands out as the best host material especially for high Yb doping concentrations. Since the lutetium and ytterbium ions have very similar ionic radii and bonding forces, the ytterbium ion can easily substitute for a lutetium ion upon doping without the overall thermal conductivity being affected due to reduced phonon scattering. This becomes a candidate material for lasers utilizing high dopant concentrations, such as thin disk or microchip lasers. Soules [5] has identified a thermal shock figure of merit, *R_T_*, where:

*R_T_* = (1 − ν) *κ K_1c_/αE* (1)

and ν is Poisson’s ratio, *κ* is the thermal conductivity, *K_1c_* is the fracture toughness, *α* is the expansion coefficient and *E* is Young’s Modulus. Soules [5] suggested using the fracture toughness instead of the strength to eliminate the large variations that are typically observed for strength values. Table 1 summarizes the estimated value for *R_T_* based on available data and highlights the superior performance of the sesquioxides compared to YAG. 

Because high power will invariably induce thermal gradients leading to thermally induced beam distortions and thermal birefringence, for high power lasers it is desirable to choose materials with low thermal expansion coefficients and a low dependence of the index of refraction on temperature, the figure of merit being a low dependence of the optical path length (OPL) on temperature. δ=dn/dT+(n−1)α. Table 2 shows that unlike the thermo-mechanical properties, fluorides and laser glass are the best in this regard and actually have a negative dependence of OPL on temperature. At very high radiation intensity the non-linear index of refraction, *n_2_*, can also lead to variation in the OPL through the system leading to beam distortion [10]. Unfortunately, the cubic sesquioxides that have good thermo-mechanical properties have a relatively high non-linear index of refraction.

**Table 1:** Properties of some cubic single crystal materials, including laser glass (LG-750) [11].

Material	*κ* (W/m·K)	*α* (ppm/K)	*E* (GPa)	*ν*	*K_1c_* (MPa·m^1/2^)	*R_T_* (10^−20^ m^2^/W)
MgO	60	11.9	294	0.186	2	28
MgAl_2_O_4_	19	5.95	258	0.2	2	20
Y_2_O_3_	13.6	7.4	173	0.307	2	14
Sc_2_O_3_	16.5	6.7	221	0.3*	2*	16
Lu_2_O_3_	12.5	5.5	178	0.3*	2*	18
Gd_3_Ga_5_O_12_	7.4	7.5	255	0.28	1.2	3.8
Gd_3_Sc_2_Ga_5_O_12_	4.9	7.3	210	0.28	1.2	2.7
Y_3_Al_5_O_12_	10.8	6.1	282	0.28	2.2	9.9
Lu_3_Al_5_O_12_	8.3	6.0	--	--	--	--
CaF_2_	9.2	19.6	110	0.25	0.5	1.6
SrF_2_	9.9	19.0	90	0.25	--	--
BaF_2_	12.1	21.1	53	0.343	--	--
LG-750 glass	0.6	13.2	50	0.26	0.45	0.30

* estimated.

**Table 2:** Optical properties including the change in index of refraction (d*n*/d*T*) and in optical path length (*δ*) across a slab with temperature and the non-linear coefficient of the index of refraction (n*_2_*, γ) [11].

Material	*n* (index)	*dn/dT* (ppm/K)	*δ* (ppm/K)	*n_2_* [11] (10^−13^ esu)	*γ* (10^−20^ m^2^/W)
MgO	1.736	19	27.8	1.61	3.89
MgAl_2_O_4_	1.72	13.2	--	1.5	3.66
Y_2_O_3_	1.78	--	--	5.33	12.60
Sc_2_O_3_	1.85	--	--	--	--
Lu_2_O_3_	1.83	--	--	--	--
Gd_3_Ga_5_O_12_	1.945	17.4	24.5	8.1	17.46
Gd_3_Sc_2_Ga_5_O_12_	1.943	10.9	17.9	--	--
Y_3_Al_5_O_12_	1.816	7.83	12.8	2.7	6.23
Lu_3_Al_5_O_12_	2.14	4.88	11.7	5.5	10.77
SrTiO_3_	2.31	--	--	26.7	48.45
ZrO_2_ (c)	2.176	--	--	5.8	11.17
CaF_2_	1.429	−10.6	−2.2	0.43	1.26
SrF_2_	1.439	−12	−3.7	0.5	1.46
BaF_2_	1.468	−14	−4.13	0.67	1.91
Sr_5_(PO_4_)_3_F	1.61262/1.61760	−5/0	0.2/5.9	1.57	4.07
LG-750	1.516	−5.1	1.7	1.08	2.99

## 2. Preparation of the Ceramics

**Figure 2:**
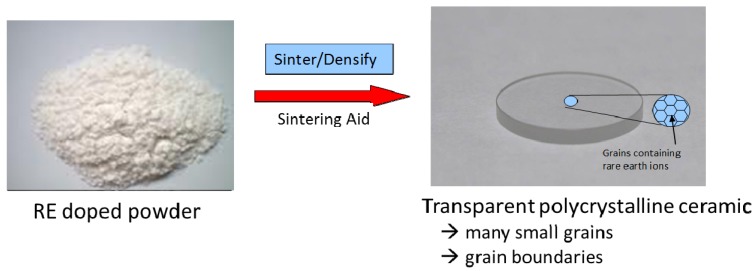
Ceramization process for converting powder into a transparent ceramic.

While the sesquioxides and YAG have excellent properties, these crystals are difficult to grow in large sizes and with high dopant concentrations using traditional high temperature melting. This is attributed to a combination of problems including compositional variations, crucible interactions, phase transitions, poor rare earth solubility. All these limit size, complexity and yield. Fortunately, the ceramization process is a low temperature route for making transparent polycrystalline ceramics (Figure 2). In this process, powder is converted into a fully dense and transparent polycrystalline ceramic material at approximately 65% of the melting point, thereby avoiding the high temperature issues associated with traditional crystal growing. The polycrystalline ceramic material looks like a single crystal, but consists of grains ranging in size from a few microns to hundreds of microns (depending on conditions) and grain boundaries that separate the grains. If the grains and grain boundaries are clean, free from pores and impurities, the ceramic can possess high transparency. This process enables higher rare earth doping, uniformity, scalability to large sizes and complex shapes, as well as making the material tougher and stronger. So, traditional limitations can be overcome with polycrystalline ceramics. 

**Figure 3:**
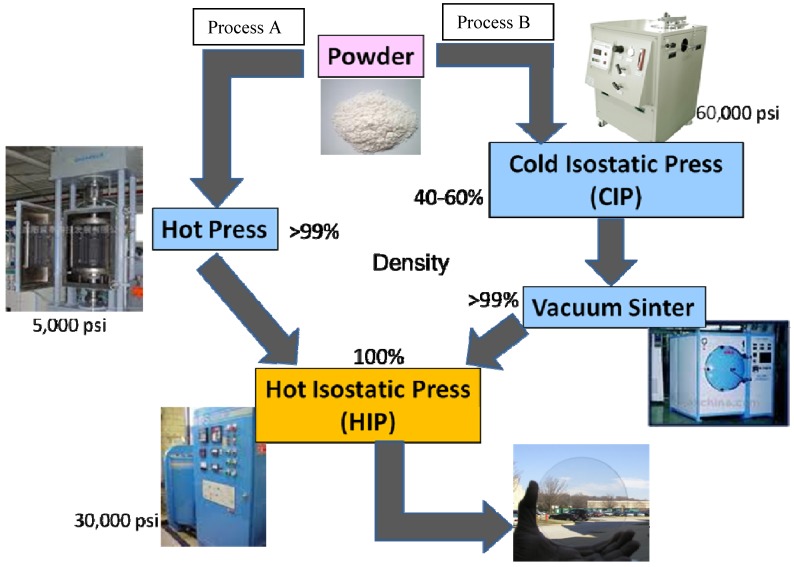
Practical fabrication of ceramics.

Figure 3 highlights two major approaches used to make transparent ceramic laser materials from powder, namely process A and B. Process A relies on hot pressing of the powder, usually in a graphite furnace, at pressures up to 5,000 psi at elevated temperatures. The product is usually 90 to 99+% dense and then further hot isostatically pressed (HIP) to full density and transparency at similar temperatures but using argon gas pressure of up to 30,000 psi to collapse any residual pores. The alternative Process B, utilizes cold forming techniques such as cold isostatic pressing (or slip casting, tape casting, extrusion, *etc*.) to make a green body usually with the addition of binders and surfactants. The green body density is typically in the range of 40–60%. Thereafter, the sample is heated in air to burn off the organics and then vacuum sintered at elevated temperatures (sometimes in the presence of hydrogen gas) to give a density of 90 to 99+%, followed by HIP to give full density and transparency. In theory it should be possible to provide full density and transparency at the end of the hot press or vacuum sintering steps in process A and B, respectively, but this is rarely done. Most groups in the literature use process B, while we use process A to make laser quality Yb:Lu_2_O_3_ ceramics.

**Figure 4:**
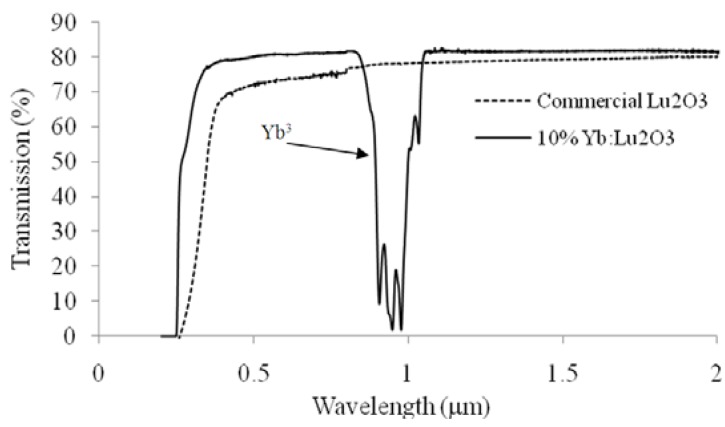
Transmission plot of the optically polished ceramics fabricated from our co-precipiated 10% Yb:Lu_2_O_3_ and commercial powders, respectively.

The quality of the starting powder is very important for making transparent ceramics. As an example, Figure 4 shows the transmission spectra for ceramic lutetia made from commercial powder and Yb doped lutetia ceramic made from high purity powder synthesized using co-precipitation (the band at 1 μm is associated with Yb dopant), respectively. The impurity content is shown in Table 3, which highlights that the co-precipitated powder and also the ceramic made using that powder have very low impurity content compared with commercial powder. 

**Table 3:** List of the impurities measured, in ppm-wt, using glow discharge mass spectrometry (EAG Lab, North Billerica, MA, USA) for the best commercial powder, NRL’s (Naval Research Laboratory) co-precipitated powder, and a transparent ceramic made using NRL’s co-precipitated powder, respectively. Only impurities greater than 1 ppm-wt are reported.

Element	Best commercial Lu_2_O_3 _powder	NRL 10% Yb:Lu_2_O_3_ powder	NRL 10% Yb:Lu_2_O_3_ ceramic
**Na**	13	<1	<1
**Mg**	<1	<1	<1
**Al**	11	<1	1.1
**Si**	88	2	<1
**P**	2.3	<1	<1
**S**	88	15	14
**Cl**	1,000	15	<1
**K**	14	<1	<1
**Ca**	9.7	<1	<1
**Fe**	1.3	<1	<1

## 3. Properties

Since a ceramic material is obviously different from a single crystal due to the presence of grains and grain boundaries, it is important to determine their impact on some specific properties. The three most important properties are optical scattering, mechanical strength and laser damage threshold. As we shall see, these properties are comparable to the single crystals, if not indeed better for the ceramics.

**Figure 5:**
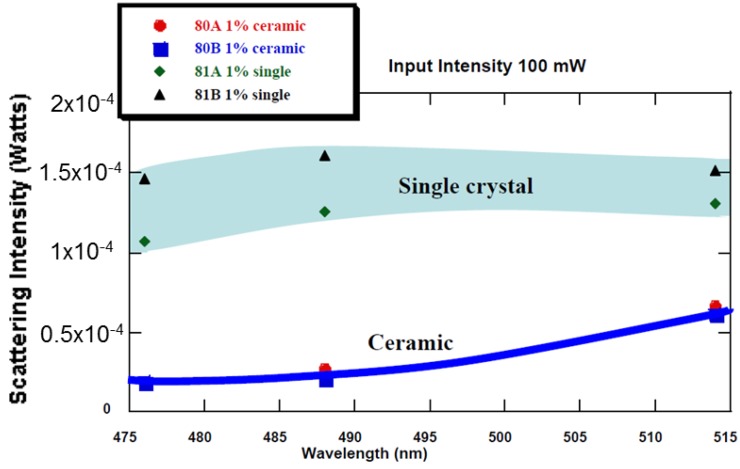
Demonstration of lower scattering loss in ceramic Nd:YAG (Quarles [12]).

Quarles [12] measured the scattering loss of high quality Nd:YAG ceramic and demonstrated that the optical scattering was in fact lower than that obtained in the single crystal (Figure 5). He attributed this to the high uniformity of the rare earth ions in the powder being maintained in the ceramics.

More recently, Feldman *et al.* [13] observed that the strength of ceramic YAG was about 1.4× higher than for single crystal YAG. Similar results have been observed by others and are listed in Table 4 and can be attributed to the increased fracture toughness of the ceramic [14,15,16]. 

**Table 4:** Strength of ceramics compared with single crystals.

Reference	Single crystal	Poly-crystalline	Poly- *vs.* single crystal
Feldman [13]	243	345	1.4×
Maziex *et al.* [14]	236	306	1.3×
R. Gentilman [15]	252	378	1.5×
G. Quarles [16]	222	287	1.3×

In ceramics, it is also well known that the strength is inversely related to the square of the grain size by the Hall-Petch equation; strength α 1/{grain size}^1/2^. Therefore, reducing the grain size will further increase the strength. Additionally, elimination of impurities and pores form the grain boundaries will improve strength and optical performance.

**Figure 6:**
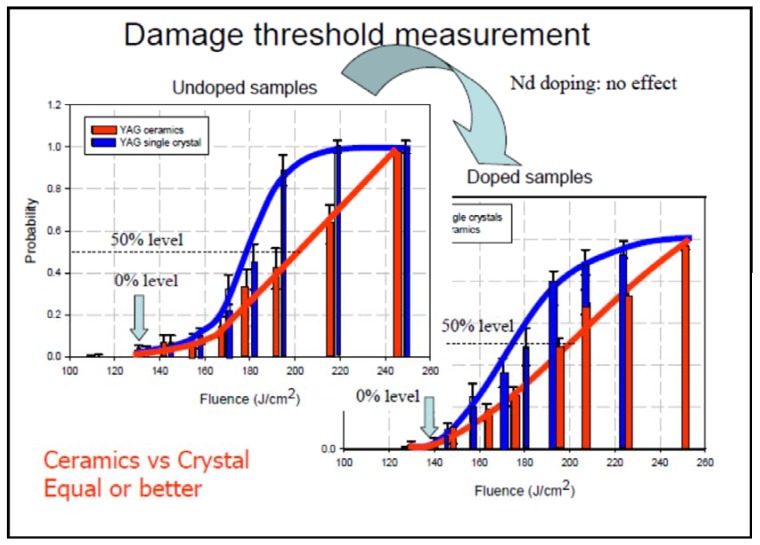
Laser damage threshold for rare earth doped and undoped YAG ceramics compared with single crystal YAG (Adapted from Ueda *et al.* [17]).

Ueda *et al.* [17] have shown that the laser damage threshold for both rare earth ion doped and undoped YAG ceramics are comparable to the single crystal, if not better (Figure 6). So, it would appear that ceramics, if fabricated correctly, possess excellent optical and mechanical properties.

## 4. History of Ceramic Laser Materials

While the physical and optical properties of ceramic YAG have improved so that it is now comparable, if not better, than single crystal YAG, the earlier ceramic lasers were of inferior quality and not necessarily made from YAG. The following highlights some key milestones in the development and demonstration of lasing using ceramic materials.

### 4.1. 1964—The 1st Ceramic Laser

Hatch *et al.* [18] were the first to demonstrate lasing in a ceramic, in this case Dy^2+^:CaF_2_. The ceramic was made by vacuum melting the tri-fluorides, grinding the product into a powder of 150 μm particle size, hot pressing the powder in vacuum and finally reducing the product to Dy^2+^ using 0.25 MeV x-rays. The ceramic product contained relatively large grains of 150 μm, implying no grain growth, and lased at liquid nitrogen temperature upon flash lamp pumping with a threshold of 24.6 J. CaO scattering centers were identified at the grain boundaries which contributed to 2% scattering loss in the visible and subsequently limited the laser performance.

### 4.2. 1973—The 1st Oxide Ceramic Laser

It took about another 9 years for the second demonstration of lasing using a ceramic [19]. This was based on 1% Nd_2_O_3_ doped Yttralox (10% ThO_2_-89% Y_2_O_3_), whereby the ThO_2_ was used to control grain growth. Greskovich and Chernoch [19] synthesized submicron powder (≤0.1 μm) using co-precipitation of oxalates and then sintered the powders under hydrogen gas at 2,170 °C. The ceramic had large sized grains (130 μm) and high scattering loss of 5 to 7 cm^−1^ attributed primarily to index inhomogeneity since the pore volume was relatively low (1 ppm) and the pores were only 1 μm. Despite this, the flashlamp pumped ceramic lased with a slope efficiency of ~0.1%.

### 4.3. 1995—1st Ceramic YAG Laser

**Figure 7:**
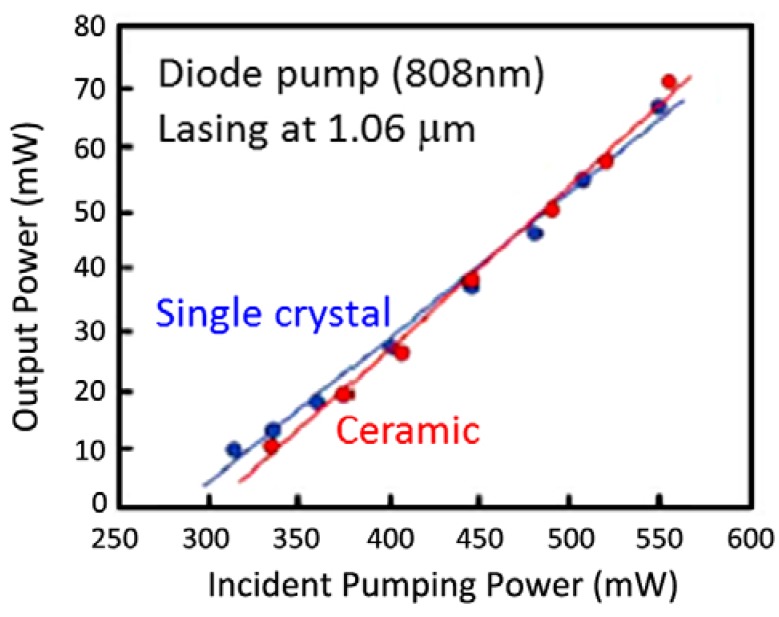
Results of the first Nd:YAG ceramic laser (Adapted from [20]).

Ikesue *et al.* [20] were the first to demonstrate lasing in YAG ceramic doped with 1.1 atomic percent Nd. They synthesized pure, submicron oxide powders (Y_2_O_3_—60 nm, Al_2_O_3_—400 nm, and Nd_2_O_3_—500 nm) with <100 ppm-wt impurity content and performed a thorough analysis of the densification dynamics via vacuum sintering. Several recommendations came out their work. Examples include the use of 320 ppm SiO_2_ sintering aid, ball milling with high purity alumina balls, spray drying the powders, and sintering at 1,700 °C to get full densification and complete conversion of the starting oxides into the YAG phase (Y_3_Al_5_O_12_) via the intermediate Y_4_Al_2_O_9_ and YAlO_3_ phases. They also recommended rapid quenching to prevent impurity phase segregation at the grain boundaries. Their process is often called “Reactive Sintering” since they started with the individual oxides and converted them to YAG during sintering. The ceramic grain size was ~50 μm, with pores less than 5 μm in diameter, a total pore volume estimated at 200 ppm and measured scattering loss of 0.9%/cm. CW (continuous wave) lasing was observed at 1.06 μm with a slope efficiency of 28% using diode pumping at 808 nm (Figure 7). The efficiency was similar to the value obtained for their single crystal sample. The ceramic also possessed very similar physical, mechanical and optical properties to the single crystal sample.

### 4.4. 2001—Nd:YAG Ceramic Laser Using Precipitated Powder

J. Lu *et al.* [21] diode pumped a 1% Nd:YAG ceramic and demonstrated lasing at 1,064 nm with an output power of 72 W and slope efficiency of 24.8%. This result was obtained using submicron 1% Nd:YAG powder that was synthesized by Konoshima Chemical Co. via a co-precipitation process followed by calcination, ball milling, slip casting and vacuum sintering. Their ceramic had a small grain size with 1 nm grain boundaries, a pore volume of only 1 ppm and birefringence similar to a single crystal. 

### 4.5. 2002—Breaking the 1 KW Output Power Barrier

**Figure 8:**
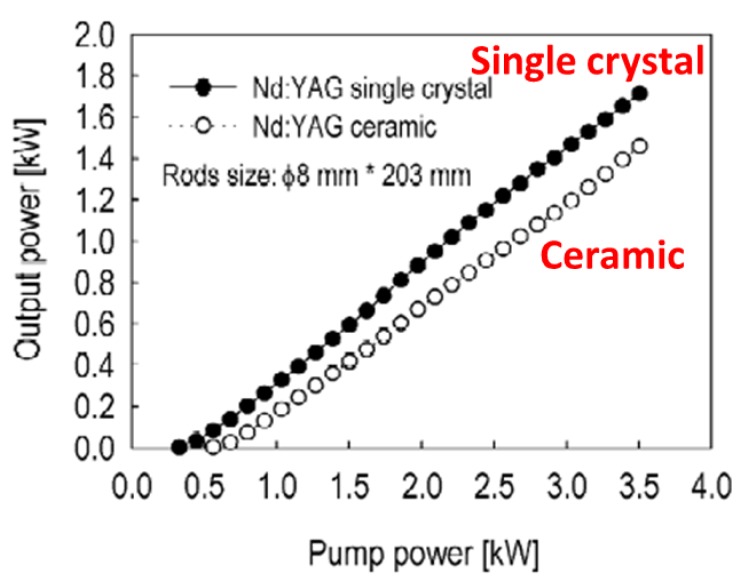
Results for first Nd:YAG laser to break 1 KW output power (Adapted from [22]).

In 2002, a group led by Ueda, in collaboration with Toshiba and Konoshima Co. (Osaka, Japan), achieved a milestone by demonstrating an output power of 1.46 KW using a Nd:YAG ceramic [22]. The slope efficiency was 42% and only slightly lower than the 49% obtained for a single crystal (Figure 8). The rod was 8 mm in diameter and 203 mm long. The high quality of the rod was attributed to improvements made in powder synthesis and sintering.

### 4.6. Microchip Lasers

**Figure 9:**
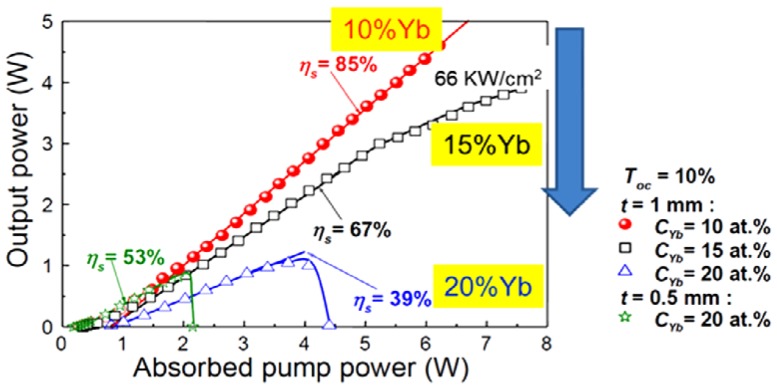
Highly doped Yb:YAG ceramic lasers (Adapted from [23]).

J. Dong *et al.* [23] have studied heavily Yb-doped YAG ceramics. For example, they demonstrated a slope efficiency of 52% for 1-mm-thick YAG ceramic doped with 20 atomic percent ytterbium ions. Heavy-doped Yb:YAG ceramic is more suitable for a thin disk laser than a single-crystal with the same Yb^3+^-ion lasants. They have also improved upon this and demonstrated up to 61% efficiency. In another example, they demonstrated a decrease in efficiency with increasing Yb content, albeit a 10% doped sample had an efficiency of 85% (Figure 9).

### 4.7. Ultrashort Pulse Lasers

**Figure 10:**
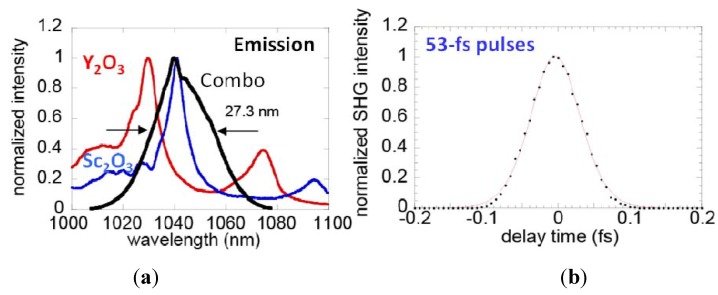
Combining a 2.5% Yb:Y_2_O_3_ ceramic behind a 1.8% Yb:Sc_2_O_3_ ceramic in a laser cavity to demonstrate (**a**) spectral broadening from nonlinear gain and (**b**) pulsed lasing with 53 fs pulses (Adapted from [25]).

Ikesue *et al.* [24] demonstrated one of the earliest mode locked lasers using a Nd:YSAG (yttrium scandium aluminum garnet) ceramic with a pulsewidth of 10 ps. The slope efficiency was 44.4% with 610 mW output power. More recently, Tokurakawa *et al.* [25] demonstrated 68 fs pulses and 540 mW average power output using a Yb:Sc_2_O_3_ ceramic with approximately 1.8 at% Yb doping. They went on to demonstrate even shorter pulses of 53 fs by combining two ceramic laser materials in front of each other (Figure 10). In this case, a 1.5 mm thick Yb:Y_2_O_3_ ceramic containing 2.5 at% Yb was placed behind the Yb:Sc_2_O_3_ ceramic. Non-linear gain and spectral broadening (Figure 10a) led to a pulsewidth of 53 fs (Figure 10b) with ~1 W average power output.

### 4.8. Non-Oxide Based Ceramic Lasers

Fluoride ceramic lasers have been demonstrated based on active color centers and rare earth ion doping [26,27]. Basiev *et al.* [26] converted LiF single crystals into a submicron-grained ceramic via hot pressing at 600 °C. Subsequent irradiation with 21 MeV electrons produced F_2_^−^ color centers which exhibit a broad absorption in the 800–1,000 nm region and also a broad emission spectrum in the 1,000–1,300 nm region. Diode pumping at 967 nm led to lasing at 1.117 μm with up to 26% slope efficiency and about 3 mW output power. This value was higher than the 18% obtained for the single crystal.

**Figure 11:**
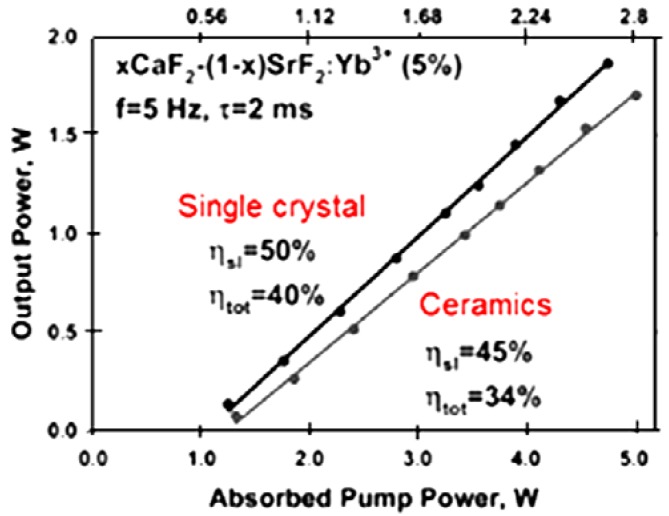
Lasing results for 5%-Yb^3+^:0.65CaF_2_-0.35SrF_2_ ceramic compared with a single crystal (Adapted from [27]).

Basiev *et al.* [27] also demonstrated pulsed lasing in 5%-Yb:0.65CaF_2_-0.35SrF_2_ ceramics made by hot pressing single crystals. They noted that the fracture toughness was increased by 75%. The ceramics lased with about 45% slope efficiency which was comparable to the single crystal value of 50%. More than 1.5 W output power was obtained (Figure 11).

**Figure 12:**
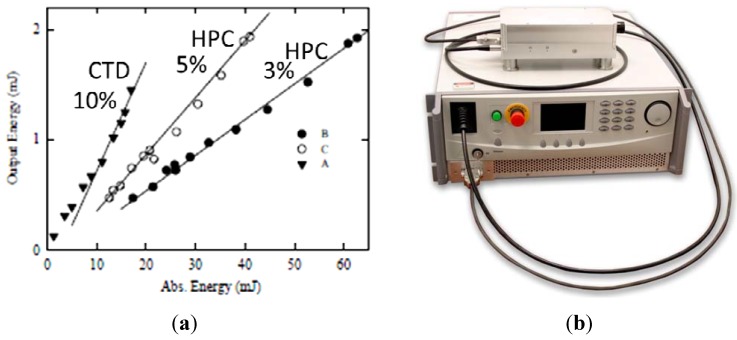
(**a**) The laser results for ceramic Cr^2+^:ZnSe made by hot pressing ceramics (HPC) and by chemical diffusion of CrSe into CVD ZnSe (CTD) (after [28]); and (**b**) a 15 W commercial source available from IPG [29].

Laser has also been observed from chalcogenide ceramics [28,29]. For example, Gallian *et al.* [28] prepared ceramic Cr^2+^:ZnSe using two techniques. One method utilized hot pressing mixtures of the powders (HPC), including 1% CrSe, and the other method involved thermally diffusing the CrSe into CVD ZnSe (CTD). The samples were pumped at 1.91 μm using a Raman shifted Nd:YAG pulsed laser. Both processes produced ceramics that lased at 2.4 μm, but the highest slope efficiencies of 10% were obtained for the thermally diffused samples compared with 5% for the hot pressed samples (Figure 12a). In fact, this technology has matured reasonably fast such that a 15 W CW laser at 2.8 μm is now commercially available from IPG [29]. The Cr:ZnSe ceramic laser is tunable from 2.05 to 2.8 μm and pumped with an Er-silica fiber CW laser (Figure 12b).

### 4.9. Composite Ceramic Lasers

**Figure 13:**
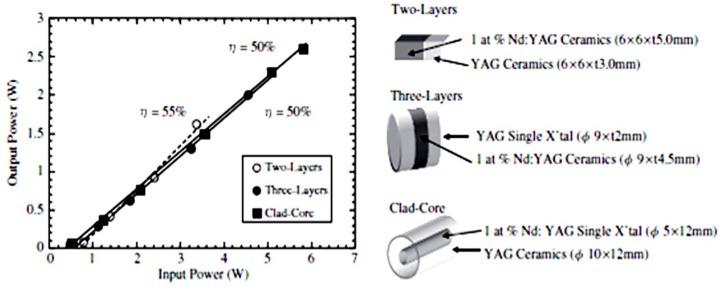
Laser performance of various types of composite elements (Adapted from [30]).

Ikesue *et al.* [30,31] demonstrated the fabrication of laser quality composite ceramics. Initial samples consisted of 1% Nd:YAG ceramics attached to undoped YAG layers as shown in Figure 13 [30], made using a layer by layer approach. The ceramic composites lased with similar efficiencies (~50%) highlighting the good quality of the samples. 

**Figure 14:**
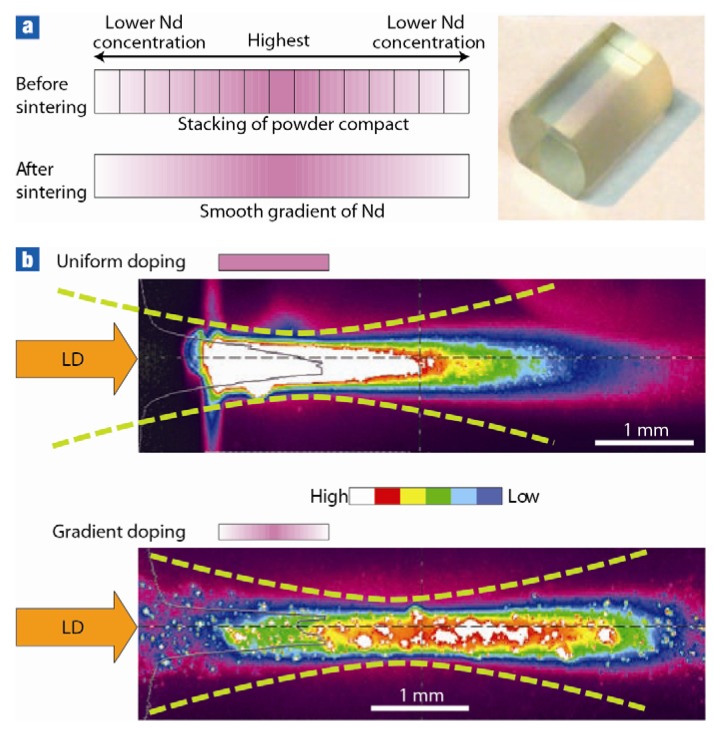
(**a**) Graded laser ceramics showing distribution of Nd ions before and after sintering and (**b**) better thermal management during lasing (Adapted from [31]).

The graded dopant profile of Nd ions in YAG samples stacked side by side before sintering is shown in Figure 14a. After sintering the stack fuses together and produces a smooth graded profile of the Nd ions that provides superior thermal management, thereby significantly reducing thermal gradients during laser operation (Figure 14b) [31]. 

**Figure 15:**
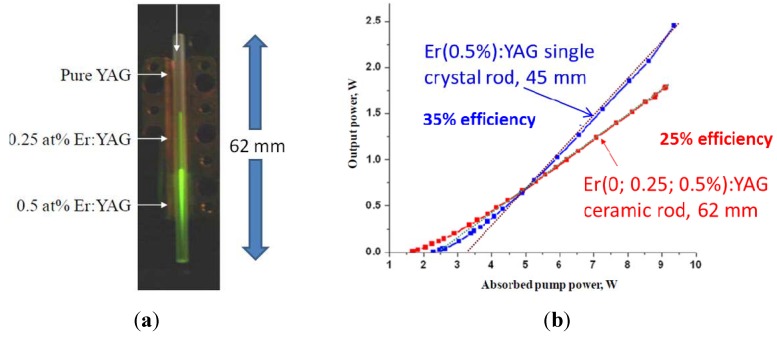
(**a**) Tape cast ceramic composite laser material and (**b**) laser result (Adapted from Messing [32]).

Messing [32] used a tape casting process to make a composite rod that lased with 25% slope efficiency and with almost 2 W output power (Figure 15). The ceramics were made by vacuum sintering of cast and stacked tapes containing the oxide powders. Binder burnout was performed prior to sintering.

### 4.10. Single Crystal Lasers from Ceramics Lasers

**Figure 16:**
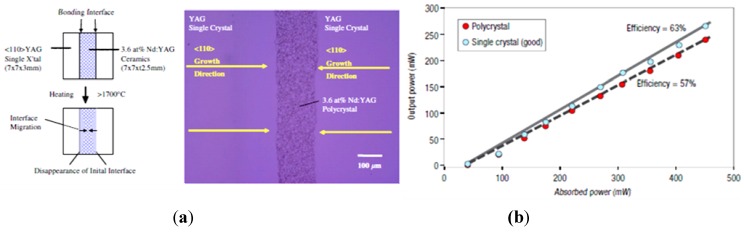
(**a**) Shows conversion of a ceramic into a single crystal by seeding a highly doped (3.6 at%) Nd:YAG with a single crystal YAG on either side and (**b**) the laser results highlighting improved slope efficiency for a single crystal produced from the ceramic, in this case containing 2.4 at% Nd (Adapted from Ikesue [30]).

Ikesue *et al.* [30] characterized the grain growth dynamics in YAG as a function of temperature and identified abnormal grain growth, also called exaggerated grain growth, above about 1,730 °C. Above this temperature, the grains can grow at very fast rates (up to 2 mm/hr). By placing a seed crystal in contact with the surface of a polished Nd:YAG ceramic, he was able to eliminate the grain boundaries, so that it appeared as though the crystal grew into the ceramic, without compromising the Nd uniformity. Basically, grain boundary migration was faster than diffusion. He went on to demonstrate this using higher doping levels of Nd (3.6 at%) (Figure 16a). In one example, the laser slope efficiency increased after conversion to a single crystal (Figure 16b).

### 4.11. Sesquioxide Lasers

**Figure 17:**
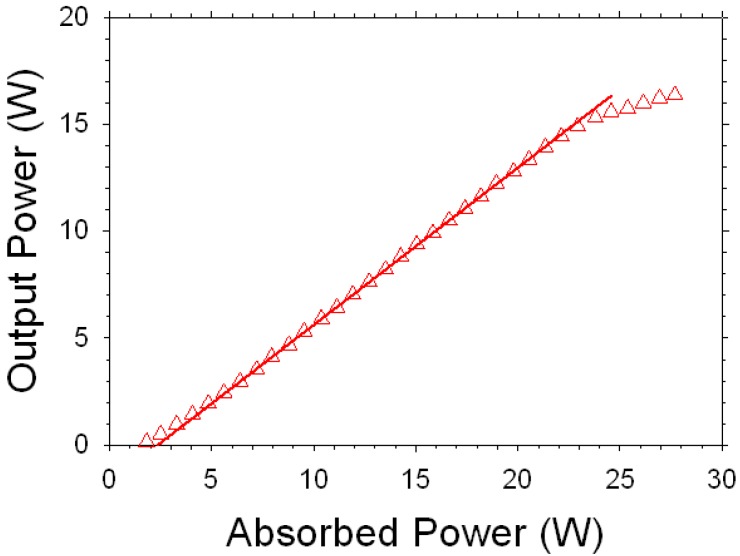
Output power *versus* absorbed power for a 10% Yb:Lu_2_O_3_ ceramic laser using a 5% output coupler. Pumping was with a diode operating at 975 nm.

Lu_2_O_3_ and the other sesquioxides (Sc_2_O_3_, Y_2_O_3_) possess higher thermal conductivities than YAG [33] which is an important property, especially for scaling to higher laser power [34]. Lu_2_O_3_ has a thermal conductivity that is predicted from fundamental principles to be almost insensitive to the Yb^3+^ dopant concentration due to negligible phonon scattering, and measurements bear this out [33,35]. Consequently, Lu_2_O_3_ would be a desirable laser host material to investigate, especially at high rare earth dopant concentrations. Therefore, we have synthesized 10%-Yb^3+^:Lu_2_O_3_ powder using co-precipitation followed by ball milling.[36] Ceramics were made by hot pressing the powder at about 1,600 °C for 2 hours. The hot-pressed samples were transparent, with densities greater than 99% of theoretical. The samples were subsequently hot isostatically pressed (HIP) at about 1,600 °C for 2 hours under an Ar gas pressure of 30,000 psi to produce fully dense and transparent ceramics. We have demonstrated lasing with an efficiency of 74% at 1,080 nm by pumping at 975 nm [37]. Figure 17 shows the laser output power *versus* absorbed power showing a maximum output power of more than 16 W using a 5% output coupler. This represents the highest output power demonstrated to-date using any Yb^3+^ doped Lu_2_O_3_ ceramic. We do observe a small roll-over in the output power at these high power levels indicating the presence of some thermal effects. Our fluorescence lifetime measurements indicate that there is no quenching in the powder, even up to 10% Yb content indicating high purity and uniformity of doping in the powder. The fluorescence lifetime of the ceramic is also comparable to the powder used to make it for up to 8% Yb doping. Thereafter, at 10% Yb doping level, the lifetime of the ceramic drops quickly indicating that there is concentration quenching, perhaps due to increased concentrations of Yb at the grain boundaries [34]. Therefore, the conclusion is to keep the Yb content at 8% or lower for the current ceramics highlighted by grains in the range of 20–50 μm. 

### 4.12. Laser Ceramics from Anisotropic Materials

Recently Taira’s group in Japan [38] showed that they could orient particles of a non-cubic material like rare-earth doped calcium or strontium flouroapatite using a high magnetic field. Once the grains are oriented and the green structure is formed, the grains remain oriented during sintering and there will not be grain boundaries between materials with different indices of refraction in the laser beam path. The group demonstrated this approach and was able to lase a small sample with a slope efficiency of 2.6%.

## 5. High Power Lasers: Towards 100KW and Beyond

**Figure 18:**
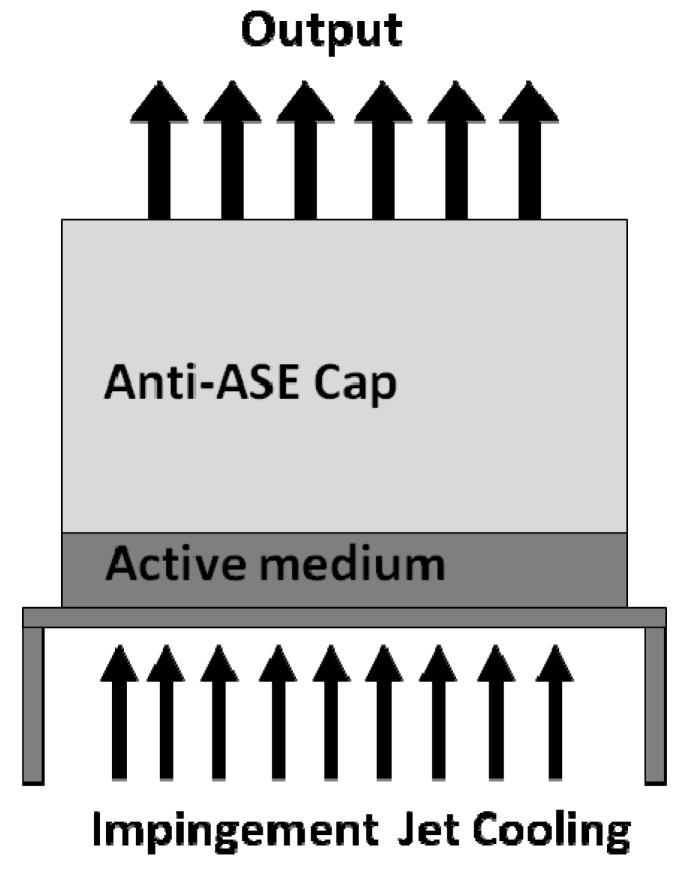
Thin disk laser using a 200 μm thick 9% Yb:YAG as the active medium and with a 1 mm thick undoped YAG cap (after [39]).

There have been some significant achievements that have led to the output power to increase from 1 KW and break the 100 KW mark. W. P. Latham *et al.* [39] reported 6.5 KW output power from a thin disk laser based on a 200 μm thick 9% Yb:YAG ceramic active medium with a 1 mm thick undoped YAG cap to mitigate thermal loading (Figure 18). Several similar thin disk lasers were combined to generate a total of >25 KW output [40].

**Figure 19:**
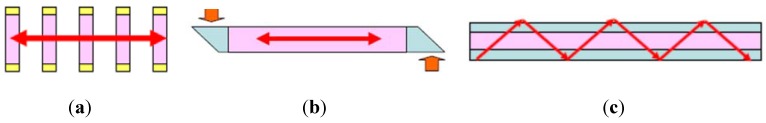
Different solid state laser configurations used for high power demonstrations: (**a**) heat capacity laser [41] (**b**) end-pumped slab laser [42] and (**c**) thinzag slab laser [43].

Three other groups have developed high power slab based lasers as shown in Figure 19 using ceramics from Konoshima Corp. For example, Lawrence Livermore Labs. [41] developed the heat capacity laser using Nd:YAG, Northrop Grumman Corp. [42] developed the end-pumped Yb:YAG slab laser, and Textron [43] developed the Thinzag (or zigzag) Nd:YAG laser.

**Figure 20:**
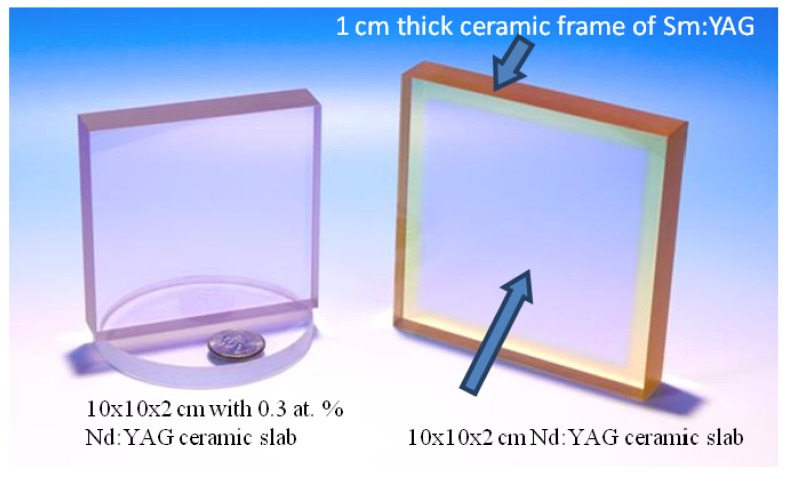
Large Nd:YAG ceramic laser slabs for the heat capacity laser (Adapted from [41]).

The solid state heat capacity laser developed by Lawrence Livermore National Lab. (LLNL) used 0.3% Nd:YAG slabs that were 10 × 10 × 2 cm. These were made by first slip casting two 1 cm thick slabs that were partially sintered, polished and then co-sintered together to increase the thickness to 2 cm. The plates were also co-sintered with 1 cm wide Sm:YAG strips around the edges to suppress ASE (Figure 20). In 2006, they demonstrated lasing with 67 KW output power for <1 s using diode pumping of 5 slabs. The slab quality was excellent, with average rms distortion at HeNe wavelength of λ/30 and surface rms ~0.5 nm. 

**Figure 21:**
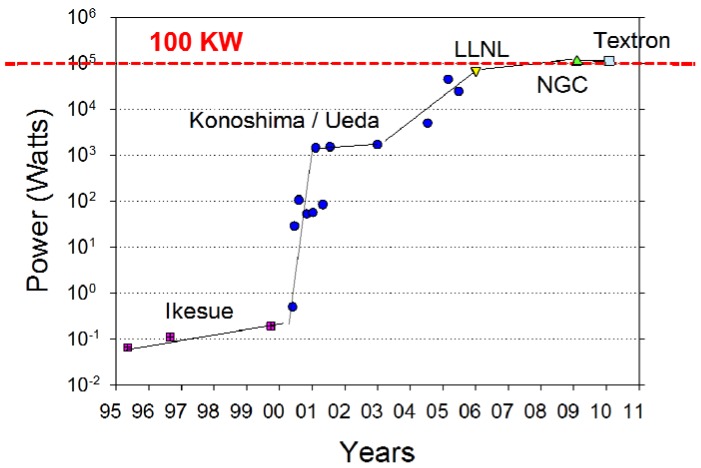
Evolution of laser output power *versus* year for YAG ceramics.

Then in 2009 Northrop Grumman Corp. (NGC) demonstrated >100 KW output power [42], followed soon by Textron in 2010 [40]. The collective evolution of output power from YAG ceramics with time is shown in Figure 21. This really highlights that the highest powers demonstrated to date originate from ceramics made by using co-precipitated powder at Konoshima Corp.

## 6. Summary

It is clearly evident that ceramic laser materials have come a long way since the first demonstration of lasing in 1964 using a Dy^2+^:CaF_2_ ceramic. It was not a rapid evolution, as noted by the fact that it took another 31 years to demonstrate lasing in ceramic Nd:YAG by Ikesue in 1995. Within another 7 years, improvements in powder synthesis and ceramic sintering enabled the 1 KW output power threshold to be broken in 2002, followed by another 7 years to break the 100 KW mark in 2009. There have been several other notable achievements that include highly doped microchip lasers, ultrashort pulse lasers, lasers made from different materials such as sesquioxides, fluorides, and selenides (e.g., for 2 to 3 μm region), composite ceramic lasers for better thermal management, and single crystal lasers derived from polycrystalline ceramics. We strongly believe that there will be many more notable achievements to follow.
